# Accreditation Standard Guideline Initiative for Tai Chi and Qigong Instructors and Training Institutions

**DOI:** 10.3390/medicines5020051

**Published:** 2018-06-08

**Authors:** Byeongsang Oh, Albert Yeung, Penelope Klein, Linda Larkey, Carolyn Ee, Chris Zaslawski, Tish Knobf, Peter Payne, Elisabet Stener-Victorin, Richard Lee, Whanseok Choi, Mison Chun, Massimo Bonucci, Hanne-Doris Lang, Nick Pavlakis, Fran Boyle, Stephen Clarke, Michael Back, Peiying Yang, Yulong Wei, Xinfeng Guo, Chi-hsiu D. Weng, Michael R. Irwin, Aymen A. Elfiky, David Rosenthal

**Affiliations:** 1 Sydney Medical School, University of Sydney, Sydney, NSW 2006, Australia; nick.pavlakis@sydney.edu.au (N.P.); franb@bigpond.net.au (F.B.); stephen.clarke@sydney.edu.au (S.C.); michael.back@sydney.edu.au (M.B.); 2 School of Life Science, University of Technology, Ultimo, NSW 2007, Australia; Chris.Zaslawski@uts.edu.au; 3 National Institute of Complementary Medicine Health Research Institute, Western Sydney University, Sydney, NSW 2751, Australia; C.Ee@westernsydney.edu.au; 4 Hanne-Doris Lang Medical Center, 20099 Hamburg, Germany; mail@hdlang.de; 5 D’Youville College, Buffalo, NY 14201, USA; kleinqpj@gmail.com; 6 Department of Family Medicine, The Catholic University College of Medicine, Seoul 06591, Korea; fmchs85@gmail.com; 7 Department of Radiation Oncology, Ajou University School of Medicine, Suwon 443749, Korea; chunm@ajou.ac.kr; 8 San Feliciano Hospital Rome, University of Chieti, 83-00166 Chieti, Italy; maxbonucci@artoi.it; 9 Massachusetts General Hospital; Harvard Medical School, Boston, MA 02115, USA; 10 Dana-Farber Cancer Institute; Brigham and Women’s Hospital; Harvard Medical School, Boston, MA 02115, USA; aymen.elfiky@sloan.mit.edu; 11 College of Nursing & Health Innovation, Arizona State University, Phoenix, AZ 85004, USA; linda.larkey@asu.edu; 12 School of Nursing, Yale University, New Haven, CT 06520, USA; tish.knobf@yale.edu; 13 Geisel School of Medicine, Dartmouth College, Hanover, NH 03755, USA; Peter.Payne@dartmouth.edu; 14 School of Medicine, Case Western Reserve University, Cleveland, OH 44106, USA; richard.t.lee@case.edu; 15 Department of Physiology and Pharmacology, Karolinska Institutet, 17165 Stockholm, Sweden; elisabet.stener-victorin@ki.se; 16 The University of Texas, MD Anderson Cancer Center, Houston, TX 77030, USA; pyang@mdanderson.org; 17 School of Acupuncture-Moxibustion and Tuina, Beijing University of Chinese Medicine, Beijing 100029, China; weiyulong@bucm.edu.cn; 18 The Second Medical College of Guangzhou University of Chinese Medicine, Guangzhou 510006, China; guoxinfeng@gzucm.edu.cn; 19 College of Tai Chi, University of East-West Medicine, Sunnyvale, CA 94085, USA; drcweng@aol.com; 20 Cousins Center for Psychoneuroimmunology, Semel Institute for Neuroscience and the David Geffen School of Medicine, University of California, Los Angeles, CA 90095, USA; mirwin1@ucla.edu

**Keywords:** Tai Chi, Qigong, standards, accreditation, certification, guideline

## Abstract

Evidence of the health and wellbeing benefits of *Tai Chi and Qigong* (TQ) have emerged in the past two decades, but TQ is underutilized in modern health care in Western countries due to lack of promotion and the availability of professionally qualified TQ instructors. To date, there are no government regulations for TQ instructors or for training institutions in China and Western countries, even though TQ is considered to be a part of Traditional Chinese medicine that has the potential to manage many chronic diseases. Based on an integrative health care approach, the accreditation standard guideline initiative for TQ instructors and training institutions was developed in collaboration with health professionals, integrative medicine academics, Tai Chi and Qigong master instructors and consumers including public safety officers from several countries, such as Australia, Canada, China, Germany, Italy, Korea, Sweden and USA. In this paper, the rationale for organizing the *Medical Tai Chi and Qigong Association* (MTQA) is discussed and the accreditation standard guideline for TQ instructors and training institutions developed by the committee members of MTQA is presented. The MTQA acknowledges that the proposed guidelines are broad, so that the diversity of TQ instructors and training institutions can be integrated with recognition that these guidelines can be developed with further refinement. Additionally, these guidelines face challenges in understanding the complexity of TQ associated with different principles, philosophies and schools of thought. Nonetheless, these guidelines represent a necessary first step as primary resource to serve and guide health care professionals and consumers, as well as the TQ community.

## 1. Introduction

Tai Chi and Qigong (TQ) are traditional Chinese mind–body exercises designed to produce functional balance in the body and emotions and to promote healing. An emerging evidence base validating the health benefits of TQ practice has resulted in a growing acceptance of these exercise arts in Western countries including Australia and the USA. Several recent studies have demonstrated that TQ can play a supportive role in management of a diversity of medical conditions to improve the quality of life of individuals with chronic diseases including arthritis [1,2], cancer [3,4,5], diabetes [6], fatigue [3,7,8] hypertension [9,10,11], chronic heart failure [12] and pain [13,14,15]. Furthermore, recent research has suggested that TQ has favorable impacts on anxiety [16], depression [17,18,19], balance [20,21,22], insomnia [23] and sleep quality [24,25], stress management [26,27], cognitive function [28,29] Parkinson’s disease [30,31], inflammation [32,33], antiviral immunity [24,34] and physical function in elderly [35,36]. 

Although research has validated the health and well-being benefits and safety of TQ, the National Center for Complementary and Integrative Health (NCCIH) identified that there may be potential risks if TQ is self-taught instead of studied with a qualified instructor, and these risks are noted especially for novice practitioners or individuals with medical conditions [37]. For example, muscular and joint pain may be exacerbated instead of relieved without appropriate instruction if TQ is not performed properly. A recent systematic review that evaluated the safety of Tai Chi in clinical trials found that several studies reported occurrences of falls and musculoskeletal pain associated with the knee, ankle, back and spine [38]. Furthermore, due to significant flaws in the reporting of adverse events in trials, information about the safety of Tai Chi is far from complete. Moreover, whereas the NCCIH recommends that individuals with medical conditions talk to their health care providers prior to commencing TQ, health care providers often do not have access to reliable information about safety due to paucity of research in this area. In addition, the review identified issues relating to difficulty of finding qualified TQ instructors in the community who have the required TQ skills and appropriate clinical training to safely deliver Tai Chi to diverse populations with varying medical conditions. 

While there are regulatory bodies overseeing the licensure qualification of practitioners of acupuncture and Chinese herbal medicine in Australia [39] and USA [40], no such body exists for the regulation of TQ. Currently, numerous private TQ training institutions in the community in Australia, China and US offer certification programs without having established accreditation standard guidelines across institutions. These local certification programs are dependent on the institution founders’ and TQ masters’ skills, knowledge and practice preferences. Thus, there is large variation in individual TQ skills and knowledge among instructors. Further, most private TQ training institutions offer TQ exercise regimen training with minimal or no foundation in biomedical and clinical science. As a result, even instructors who are well known in their local community with a high level of TQ skills, may have insufficient medical knowledge to address adequately the potential risk and benefits of TQ as they train and work with students and/or patients and unique concerns related to clinical practice and specific medical conditions. Furthermore, many TQ instructors may not be able to recognize whether an unforeseen adverse event is due to the practice of TQ. To date, there are no published accreditation standard guidelines for TQ instructor training that recommend integration of TQ practice and Western health care. In recognition of this major limitation, the Medical Tai Chi and Qigong Association (MTQA), a non-profit, credentialing organization, was established.

The aims of MTQA are: 

(1) to form and maintain an accreditation committee to develop and review accreditation standard guidelines for TQ instructors and training institutions within the context of integrative health care;

(2) to establish and support *certified medical TQ instructors* (CMTQI) as emergent health care professionals to meet a work force need; and

(3) to inform the medical community as well as the general public, as to the credentialing and scope of practice of clinical TQ providers and the differentiation between traditional TQ instructors and those certified as medical TQ practitioners.

In this report, the term *Tai Chi and Qigong instructor* (TQI) refers to individuals who teach traditional Tai Chi and/or Qigong as an art or for health promotion. We define *certified medical TQ instructors* (CMTQI) as qualified individuals who teach or prescribe Tai Chi and/or Qigong with the intention of improving specific health outcomes, and as an adjunct to standard medical care provided by the patient’s usual medical practitioners. To become a CMTQI, a traditionally-trained Tai Chi and Qigong instructor is required to complete additional clinical training and consumer/public safety awareness. This report proposes accreditation standard guidelines for CMTQI certification.

## 2. Methods

Initially, an international TQ expert advisory committee was assembled by invitation to develop the *Accreditation Standard Guidelines Initiative for Tai Chi and Qigong Instructors and Training Institutions* (ASGITQIT). Communication was via email (May 2017–March 2018). Development of the *Accreditation Standard Guidelines for Tai Chi and Qigong Instructors and Training Institutions* (ASGITQIT) followed a structured and predefined consensus process, which included a pre-workshop online discussion (May–October 2017), large group discussions and an adapted *world café* methodology that is a simple, effective, and flexible format for facilitating large group dialogue (May–October 2017), a consensus workshop (November 2017 Society for Integrative Oncology (SIO) conference in Chicago, IL, USA), and email communication (June 2017–March 2018) utilizing written comments to finalize the document. Participants from multiple backgrounds were involved in the consensus process for this ASGITQIT to warrant internal and external validity in the recommendations. Participants of the workshop had the following backgrounds: seven experts in TQ with experience in both TQ practice and TQ research (one from Australia, one from Canada, one from Korea, two with a health professional background living in the USA, and two academics from the USA). Of the seven participants (BO, GP, MC, RL, DR, PK, and LL), three have experience in TQ research (BO, PK, and LL). Expert involvement was further broadened by the inclusion of twenty international Complementary Medicine (CM) research experts (CE, CZ, TN, PP, ESV, RL, WC, MC, MB, HDL, NV, FB, SC, MB, PY, YW, XG, AE, AY, and MRI) who did not participate in the workshop, but who contributed to the consensus process as advisory committee members. The consensus process was finalized after feedback from all workshop participants and the external review experts.

### Advisory Committees

The MTQA advisory committee consists of four expert groups from Australia, Canada, China, German, Italy, Korea, Sweden, and USA: Medical Advisory committee members, Tai Chi and Qigong Advisory Committee, Science and Education Committee, and Friends of Tai Chi and Qigong Committee.

(a) Medical Advisory Committee members: Health professionals including medical doctors who assist with developing a clinical medicine educational program.

(b) Tai Chi and Qigong Advisory Committee Members: Tai Chi and/or Qigong masters who participate in developing a core Medical Tai Chi and Qigong program and assess the applicant’s Tai Chi and/or Qigong practical skills.

(c) Science and Education Committee members: Academic researchers who are involved in Tai Chi and Qigong scholarly activities and provide up-to-date research on the use of Tai Chi and/or Qigong in medicine.

(d) Nominations from Tai Chi and Qigong Committee members (Consumer representative): Individuals attending Tai Chi and Qigong class in their community or expressed interest in TQ activities who participated in process of developing the guidelines of MTQA especially from a consumer’s perspective. In addition, Public Safety officers were invited to oversee consumer protection.

## 3. Results

The results of this collaboration are codified as Association policies and procedures and standards for achieving certification as a Medical Tai chi Qigong Instructor. Detailed definition of standards of practice provides the foundation for developing entry level and continuing educational curriculum. Four levels of certification are defined. Level I connotes basic entry-level skill. Level II connotes more advanced clinical skills. Level III connotes advanced evaluative skills. Level IV connotes mastery skills and leadership that advance the discipline in at least two or more of the following areas: clinical application, education, research, and/or service (see appended document for specifics).

### 3.1. Assessment protocol for the Certificate in Medical Tai Chi and/or Qigong Instructor (CMTQI) 

#### 3.1.1. Current Tai Chi and/or Qigong Instructors without Health Professional Academic Degrees

**Figure 1:**
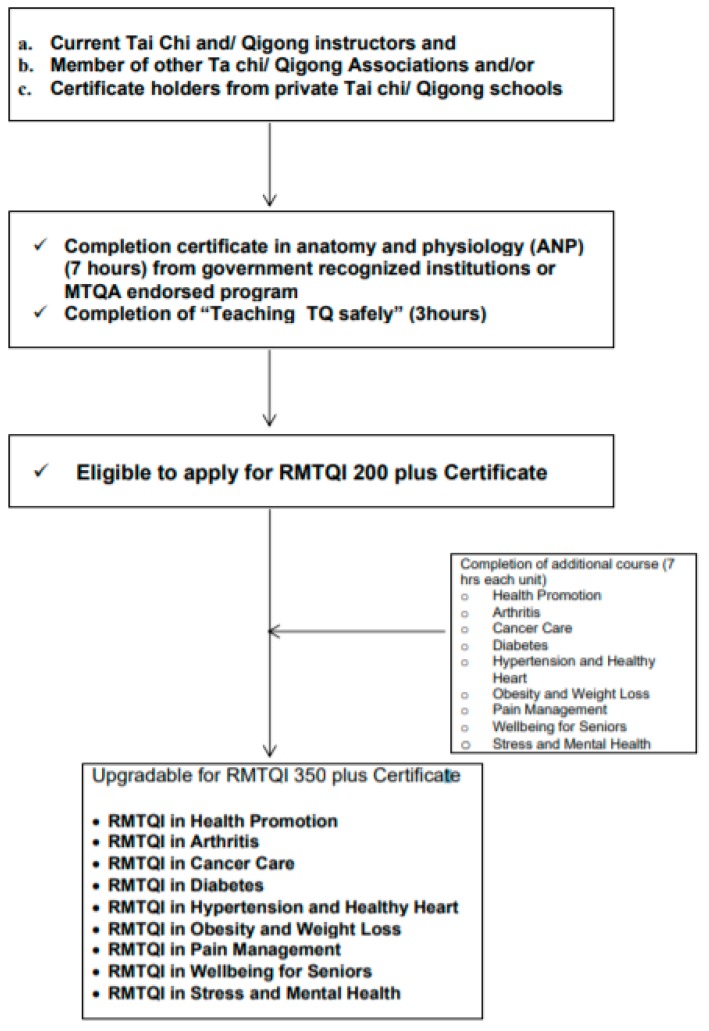
Current Tai Chi and/or Qigong instructors without health professional academic degrees.

To be eligible to register as a CMTQI in health promotion, current traditional Tai Chi and/or Qigong instructors without health professional academic degrees are required to complete anatomy, physiology, and Medical Concentration (APM) course or courses offered by tertiary institutions or organizations as approved by the MTQA Certification Advisory Committee. Instructors awarded the CMTQI in health promotion are eligible to apply the specialty designations status based on life experience or after completion of additional designation courses described below in Figure 1.

(For example, Certified clinical Tai Chi and Qigong instructors who complete the cancer medicine unit in addition to APM are eligible to get the award the CMTQI in Cancer Care.)

#### 3.1.2. Current Tai Chi and/or Qigong Instructors Who Have Work Experience at Hospitals or Health Care Institutions but No Health Professional Academic Degrees

**Figure 2:**
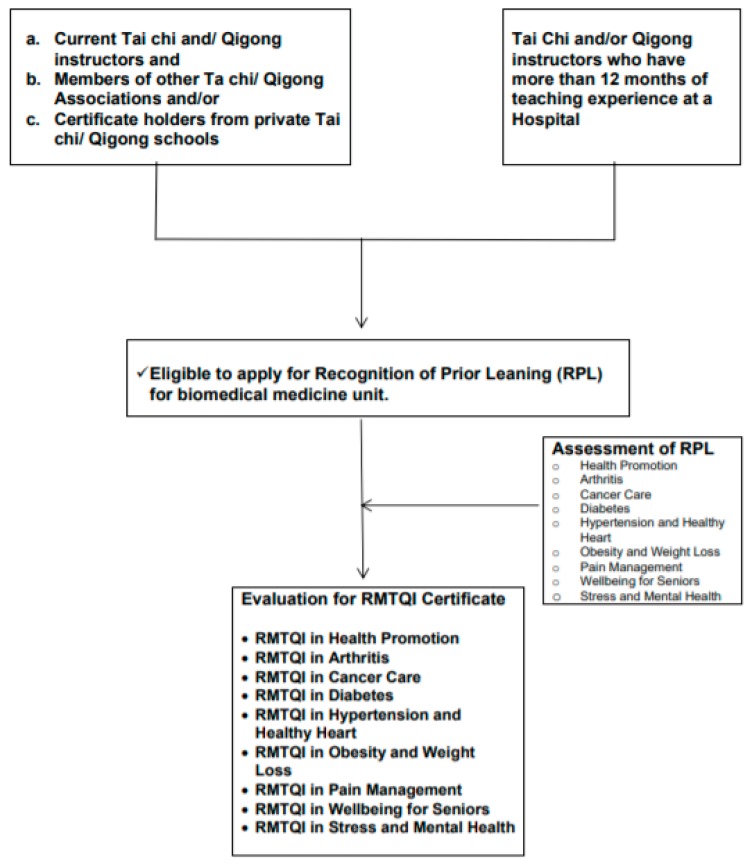
Current Tai Chi and/or Qigong instructors who have work experience at Hospitals without health professional academic degrees.

Current traditional Tai Chi and/or Qigong instructors who have been delivering Tai Chi and/or Qigong programs at Hospitals or health care institutions for more than 12 months but who do not have health professional academic degrees are eligible to apply for the CMTQI subject to recognition of prior learning (RPL). Applicant will be asked to submit three case studies as part of RPL assessment. A special designations award will be decided by the MTQA/CMTQI Certification Board subject to RPL. Other categories of specialty designations can be applied for only after completion of additional designation courses as described below in Figure 2.

(For example Traditional Tai Chi and Qigong instructors who have delivered Tai Chi programs for groups diagnosed with hypertension, may get the special designation award CMTQI in Hypertension and Healthy Heart subject to RPL without completion of ANP and biomedicine units. Further, if an instructor has received the special designation of CMTQI in Hypertension and Healthy Heart, and would like to register for another category of special designation, the instructor is required to complete additional biomedicine units relevant to the special designation applied for.)

#### 3.1.3. Current Traditional Tai Chi and/or Qigong Instructor with Health Professional Academic Degrees

**Figure 3:**
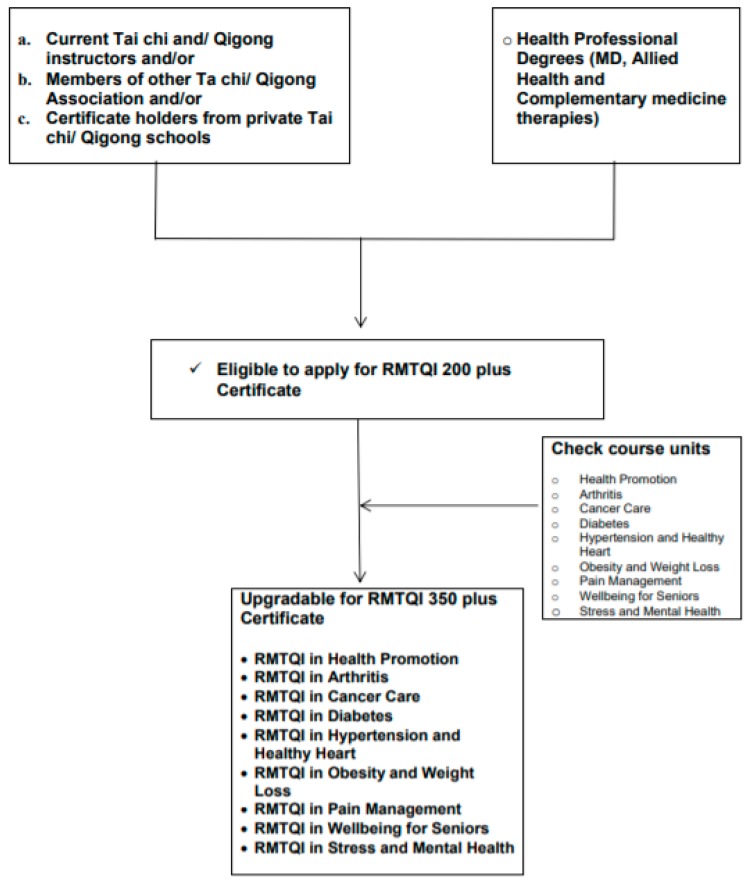
Current Tai chi and/or Qigong instructors with health professional academic degrees.

Current traditional Tai Chi and/or Qigong instructors who have health professional academic degrees such as a medical degree, allied Health (nurse, midwife, physiotherapy, etc.) and a degree in complementary medicine therapies (acupuncture, Chinese medicine, naturopathy, etc.) are eligible to apply for the award of multiple categories of special designations. The CRMTQI will be awarded after evaluation of relevant transcripts or current certificate of health care registration issued by relevant government bodies as described in Figure 3).

(For example, Traditional Tai Chi and Qigong instructors who have received degrees of medicine, or nursing or Chinese medicine are eligible to apply for the award of multiple categories of special designation of CRMTQI without completion of additional ANP and biomedicine units. However, the multiple special designations of CRMTQI will only be awarded by the MTQA/CRMTQI Certification Board after evaluation of supporting documents.)

#### 3.1.4. Current Tai Chi and/or Qigong Instructor without Supporting Documents

**Figure 4:**
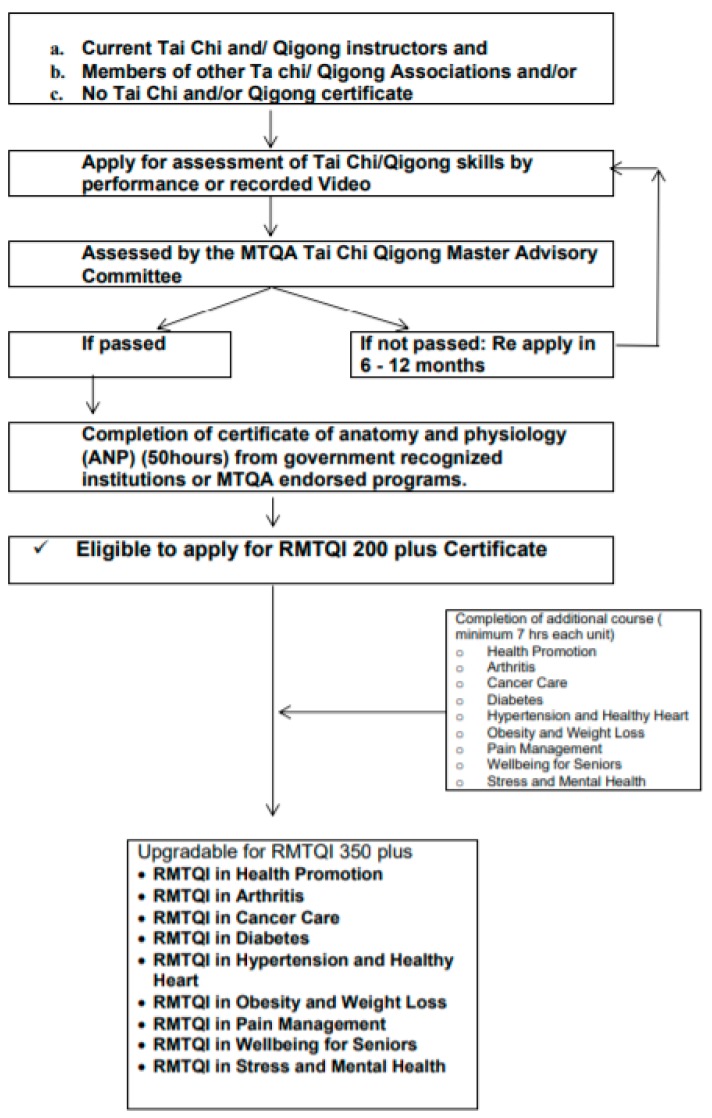
Current Tai Chi and/or Qigong Instructors without supporting documents.

Current traditional Tai Chi and/or Qigong instructors who have Tai Chi and/or Qigong teaching experience in the community of more than 12 months but have no supporting document (certification of competition of Tai Chi and/or Qigong training from the institutions of their Tai Chi and/or Qigong masters) are also eligible to apply for the CMIQI subject to recognition of prior learning (RPL). In determining RPL, an applicant’s Tai Chi and/or Qigong skills will be assessed by the member of Tai Chi and Qigong Advisory Committee of MTQA either in person or by recorded video tape. Once an instructor’s Tai Chi and/or Qigong skills are assessed as satisfactory by the member of Tai Chi and Qigong Advisory Committee of MTQA, then the applicant must complete an anatomy and physiology (ANP) course to be eligible to receive the CMIQI in health promotion. Once the CMIQI in health promotion is awarded, an instructor is eligible to apply for further specialty designations following completion of additional courses relevant to special designations as described below in Figure 4.

#### 3.1.5. Accreditation Guidelines for Tai Chi and Qigong Training Institutions

**Figure 5:**
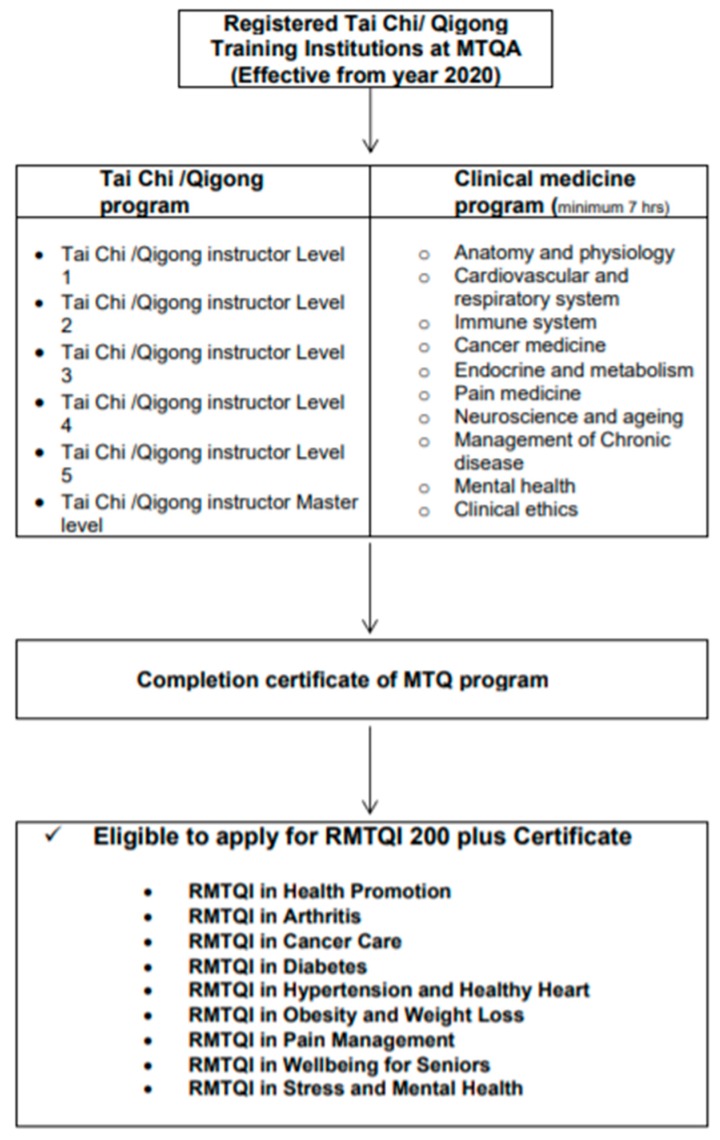
Accreditation Guidelines for Tai Chi and Qigong Training Institutions.

There are no Tai Chi and/or Qigong training institutions currently offering a combination of both Tai Chi and Qigong training programs and biomedicine programs. Therefore, the MTQA has developed guidelines for a Medical Tai Chi/Qigong program for Medical Tai Chi and Qigong Training Institutions based on MTQA standard accreditation guidelines as described in Figure 5. The MTQA encourages Institutions to integrate Tai Chi/Qigong and biomedicine programs to enable graduates to be eligible for Medical Tai Chi and Qigong Instructor Certification after completion of their course.

Tai Chi and Qigong Program Designed to Learn Practical Psychomotor Skills of Tai Chi/Qigong

- Tai Chi/Qigong Instructor Level 1 (minimum 200 h of practical training plus 7 h of Qi theory)
- Tai Chi/Qigong Instructor Level 2 (minimum 350 h of practical training plus 7 h of scientific evidence)
- Tai Chi/Qigong Instructor Level 3 (minimum 500 h of practical training plus 7 h of application of Qi in medicine)
- Tai Chi/Qigong Instructor Level 4 (minimum 1000 h plus 7 h of Qi for longevity and prevention of diseases)
- Tai Chi/Qigong Instructor Level 5 (minimum 1500 h plus 7 h of Qi, mind and spirit)
- Tai Chi/Qigong Instructor Master level by acclamation of Advisory Board

Biomedicine Continuing Education Designed to Promote an Understanding of the Human Body and Improve Medical Knowledge in General and Specific to Certification in Designated Clinical Areas

◦ Anatomy and physiology (minimum 7 h)
◦ Medical concepts and introduction to clinical pathology (minimum 7 h)
◦ Effective class management, Ethics and Risk Management (minimum 7 h)
◦ Cardiovascular and respiratory system (minimum 7 h)
◦ Immune system (minimum 7 h)
◦ Cancer medicine (7 h)
◦ Endocrine and metabolism (minimum 7 h)
◦ Pain medicine (minimum 7 h)
◦ Neuroscience and ageing (minimum 7 h)
◦ Management of Chronic disease (minimum 7 h)
◦ Mental health (minimum 7 h)

## 4. Discussion

With growing scientific evidence of the health and wellbeing benefits of TQ and in recognition of the potential benefit in the management of chronic disease and rehabilitation, several TQ organizations^-^ and private training institutions have established and developed their own accreditation standard guidelines in China [41,42] and some Western countries [43,44]. It appears that most TQ organizations and private training institutions’ accreditation guidelines are aimed towards health promotion of the general public rather than the health of individuals with medical conditions. Hence, a large percentage of existing accreditation guidelines are based on the instructor’s TQ skills, performance and competency and do not address the value of the medical knowledge for instructors to deliver TQ to individuals with medical conditions. Recently, several clinical practice guidelines in Western medicine embraced mind–body therapy techniques including TQ in the management of several chronic diseases following increasing evidence of the health and wellbeing benefits of TQ. Nonetheless, there is no reliable information or database available to health care providers to search for or identify qualified TQ instructors in the community to refer clients who have health and wellbeing issues. To fill this gap and in response to the demand of health care providers to identify qualified TQ instructors, we formed the committee members, established the Medical Tai Chi and Qigong Association (MTQA) and developed the accreditation standard guidelines initiative for TQ instructors and training institutions. To the best of our knowledge, this is the first accreditation standard guidelines initiative for TQ instructors and training institutions developed in collaboration with health professionals, integrative medicine academics, Tai Chi and Qigong master instructors and consumers from several countries including Australia, Canada, China, Germany, Hong Kong, Italy, Korea, Sweden, and USA. The primary significance of current accreditation standard guidelines is the recommendation of basic clinical medicine training to TQ instructors, as well as the TQ skills and competence to deliver TQ safely to prevent potential adverse events. Further, the guidelines also aim to encourage communication between TQ instructors and health care providers. Hence, prior to applying for the TQ instructor qualification, evidence or proof of basic medical knowledge is mandatory if applicants wish to deliver or prescribe TQ to individuals who have medical conditions. Furthermore, the guidelines recommend TQ training institutions include the biomedicine program as part of their certification training program.

Prior to preparation of the present accreditation standard guidelines, two TQ consensus papers were reported. The first Qigong and Tai Chi consensus report was published in 2005 after meeting with a group of international Qigong and Tai Chi experts in the US [45]. It attempted to address the health and wellbeing benefits of TQ and its implication in health care [45]. In the panel meeting, TQ expert participants mainly highlighted the essential components of the TQ program contents and structures including body movements, postures, breathing and meditation practice and self-administered massage. The guidelines also identified and made a recommendation of basic training requirements and knowledge, skills, and abilities for TQ instructors to lead classes and practices sessions. However, it did not acknowledge the significance of medical knowledge for TQ instructors or the development of basic biomedical education for TQ instructors although it discussed the implication of TQ in the US health care system. The latest Qigong consensus guideline published in 2017, “Qigong for cancer care programming”, identified and addressed the magnitude of required knowledge of cancer medicine in addition to Qigong theory in Traditional Chinese medicine [46]. Neither consensus report addressed the value of TQ instructors’ medical knowledge to deliver TQ in a safe manner and prevent injuries. The safety of TQ before efficacy is a major concern for the medical community even though it is considered a low impact exercise and current evidence supports its efficacy for health and wellbeing. Thus, the current guideline recognizes the value of medical knowledge and it is recommended as a mandatory requirement for qualified instructors in addition to TQ skills.

In relation to the safety of TQ, a systematic review conducted in people with fibromyalgia syndrome found that the main reason for drop out (3.1%) in clinical trials was due to adverse events such as increased pain and muscle inflammation [47]. A recent systematic review conducted on TQ for Parkinson’s disease also identified minor adverse events with Tai Chi such as pain, falls, and dizziness in small populations [30]. Case studies examining adverse effects resulting from practicing Qigong in people with psychiatric histories reported that inappropriate practice of Qigong may induce abnormal psychosomatic responses and even mental disorders [48,49], in contrast to several recent studies that demonstrated that Qigong can improve both physical and psychological conditions [50,51].

Several issues related to the current project were also raised by committee members of MTQA during the consultation process. Here, we report some of them and how they were resolved among members.

### 4.1. Why Do Tai Chi/Qigong Instructors Need to Know Anatomy and Medical Knowledge? 

The MTQA recommends to instructors, who will be delivering programs to patients, to undertake a basic clinical medicine course so that they are better equipped to target Tai Chi and Qigong to suit the patient’s needs. This recommendation was initiated from health care practitioners in the community who desire to refer their patients to qualified Tai Chi and Qigong instructors.

### 4.2. How Many Hours of Biomedical Education Are Appropriate for TQ Instructors?

Currently, there were no existing guidelines to recommend appropriate biomedical education programs for TQ instructors. Most TQ Associations worldwide issue the certificate to their instructors based on TQ practical competencies. Initially, our Medical advisory Committee (*n* = 5) and Scientific committee members (*n* = 5) recommend twenty hours of biomedical education for each unit. However, during the consultation process, TQ advisory committee members (*n* = 7) who are running TQ schools in the community and students (*n* = 10) suggested that it will limit the TQ instructors joining the association due to rigid requirements. Considering this issue, Scientific committee members (*n* = 4) who have had clinical trial experience and TQ and TQ advisory committee members reached an agreement to recommend seven hours of each biomedical unit provisionally over the next three years.

### 4.3. Tradition Holds That There Are Thousands of Forms of Qigong That Have Been Recognized in the World and in That Spirit, More Are Likely to Emerge. It Seems to Me to Be Hubristic and Contrary to the Essence of Qigong That any Group Might Try to Establish Its Own Set of Standards That Would Stifle This Dynamic Body of Practices 

We believe that we have considered the fact that there are a multitude of Tai Chi and Qigong forms that originate from Daoist philosophy and practice, religion, martial art and traditional Chinese medicine. Thus, we respect each school of traditional Tai Chi (e.g., Chen, Yang and Sun style) and Qigong (e.g., Daoist and Shaolin Kung fu) school of thoughts and philosophy. We propose to keep tradition and accept the value of their traditional Tai Chi and/or Qigong, rather than proposing or imposing a rigid standard form of Tai Chi and Qigong. We aim to provide basic guidelines to Tai Chi and Qigong Masters in the community to evaluate students who would like to be instructors so that they provide effective and safe Tai Chi and Qigong. 

### 4.4. How Can We Identify Who Is a Good Practitioner and Who Is Not? 

There are several Tai Chi and Qigong Associations worldwide (e.g., American Tai Chi and Qigong Association, Australian Tai Chi and Qigong Association, British Tai Chi and Qigong Association, Canadian Tai Chi Association, Chinse Health Qigong Association, Society of, International Wu Style Tai Chi Chuan Federation, World Tai Chi Federation in Taiwan, etc.). We will collaborate with the National or International Tai Chi and Qigong Associations to identify Tai Chi Masters and send out invitation letters to join our association.

### 4.5. Have the Opinions of Tai Chi/Qigong Masters from China Been Integrated into This Guideline?

Several emails were sent to six academic institutions in China and to date only one response has been received. Currently, consultation is being directed to individuals of Tai Chi and Qigong Associations in China (Chinse Health Qigong Association, Society of, International Wu Style Tai Chi Chuan Federation, World Academic Society of Medical Qigong, and World Tai Chi Federation in Taiwan) in the hopes of gaining their expertise and prospective. 

## 5. Limitations

One limitation of the proposed guideline is that we have had limited collaboration opportunities with TQ masters and researchers in China during the process of developing guidelines due to language barriers. However, more that 50% of our committee members, particularly in the TQ master advisory committee, that participated in the process of developing guideline had a Chinese cultural background. Hence, we feel that the proposed guidelines can be applicable to both Western and Asian countries. In addition, considering that the current project was initiated by volunteers in the committee member of MTQA, we plan to seek funding to evaluate the guidelines from TQ communities, health professionals, consumers and policy makers. 

## 6. Implication

The proposed guidelines based on an integrative health care approach have several implications. First, these accreditation guidelines can serve as a foundational resource to guide health care professionals to identify qualified TQ instructors in their community. Further, these guidelines will provide information to health care providers on the requirements of training for TQ instructors (e.g., duration, hours of training program, and content and evaluation parameters). Furthermore, the accreditation process has value to serve individuals in community to self-identify qualified TQ instructors when they consider join TQ classes to manage their health and well-being, which have significant implication as TQ training institutions consider developing TQ training program for future instructors. These accreditation guidelines suggest that training programs should include both TQ skills, basic medical knowledge and public safety ethics. Thus, instructors graduating from an accredited training program will have the ability to deliver TQ in a safe manner, particularly, to individuals who have medical conditions. Finally, the current guidelines can be a useful resource to enhance TQ instructor-medical doctor communication and encourage research collaboration opportunities between TQ instructors and researchers.

## 7. Conclusions

This accreditation standard guideline initiative for TQ instructors and training institutions was developed in collaboration with multiple stakeholders (health professionals, integrative medicine academics, Tai Chi and Qigong master instructors and consumers) from several countries. Committee members agree on the value of medical knowledge and understand the complexity of the nature of traditional TQ style and principles inherited from a multiplicity of TQ masters and schools. Hence, instead of proposing a standard form of TQ, the current guideline suggests TQ instructors to enhance their basic medical knowledge in addition to TQ skills to deliver TQ in a safe manner. It also encourages TQ instructors to communicate with health professionals to further optimize the health and wellbeing of patients who seek TQ to manage their medical conditions. 

## Supplementary Materials

The following are available online at http://www.mdpi.com/2305-6320/5/2/51/s1.

## Medical Advisory Committee Members

David Rosenthal, MD, Harvard Medical School (HMS), USA; Albert Yeung, MD, Massachusetts General Hospital & HMS, USA; Aymen Elfiky, MD, M.Sc., MBA, Dana-Farber Cancer Institute, HMS, USA; Roger Schneider, MD, Village of Healing and Wellness, Canada; Paul Lam, MBBS, Tai Chi for Health Institute, Australia; Fran Boyle, MBBS, PhD, Mater Hospital, Sydney Medical School (SMS), Australia; Stephen Clarke, MD, PhD, Royal North Shore Hospital, SMS, Australia; Nick Pavlakis, MBBS, PhD, Royal North Shore Hospital, SMS, Australia; Carolyn Ee, MBBS, Grad Cert Med Acup, PhD, NICM, Western Sydney University, Australia; Danforn Lim, MBBS, University of Technology Sydney, Australia; Judith Lacey, MD, Head of Supportive Care, Lifehouse, Royal Prince Alfred Hospital, Sydney, Australia; Kyung Ju Lee, MD, Korea University Medical School, Korea; Mison Chun, MD, Department of Radiation Oncology, Ajou University School of Medicine, Suwon, Korea; Whanseok Choi, MD, Department of Family Medicine, Seoul St. Mary’s Hospital, Catholic University of Korea; Massimo Bonucci, MD, San Feliciano Hospital Rome, University of Chieti, Italy; Hanne-Doris Lang, MD, Medical Center, Hamburg, Germany; Michael Back, MBBS, MBA, Royal North Shore Hospital, SMS, Australia; Richard Lee, MD, Case Western Reserve University, Cleveland, OH, USA; Yulong Wei, MD, PhD in TCM, Executive Vice Director of Academic Committee of World Academic Society of Medical Qigong (WASMQ), Beijing University of Chinese Medicine, Beijing, China; Michael R. Irwin, MD, Director, Mindful Awareness Research Center, Cousins Center for Psychoneuroimmunology, Semel Institute for Neuroscience, David Geffen School of Medicine at UCLA, CA, USA.

## Science and Education Board Members

Linda Larkey, PhD, Arizona State University, USA; Tish Knobf, PhD, RN, School of Nursing, Yale University, USA; Pei-Shiun Chang, PhD, RN, Indiana University, USA; Chris Zaslawski, PhD, University of Technology Sydney (UTS); Byeongsang Oh, OMD, PhD, Royal North Shore Hospital, SMS, Australia; Michael de Manincor, PhD, Yoga Institute, Australia; Shuai Zheng, PhD, UTS Sydney; Myeong Soo Lee, PhD, Korea Institute of Oriental Medicine, Korea; Peiying Yang, The University of Texas, MD Anderson Cancer Center, TX, USA; QingChuan Hu, PhD candidate, Beijing University of Chinese Medicine Beijing, China; XinFeng Guo MD, M.P.H., PhD. Guangdong Provincial Hospital of Chinese Medicine, The Second Medical College of Guangzhou University of Chinese Medicine, China.

## Tai Chi Qigong Advisory Committee Members

Penelope J. Klein, PT, EdD, D’Youville College, Buffalo, NY, USA; Linda Larkey, PhD, Arizona State University, Phoenix, AZ, USA; Byeongsang Oh, PhD, Royal North Shore Hospital, Sydney Medical School, Australia; Shuai Zheng, PhD, University of Technology Sydney; Paul Lam, MD, Tai Chi Master, Tai Chi for Health Institute, NSW, Australia; Peter Payne, Master Instructor, Geisel School of Medicine, Dartmouth College, USA; George Picard, Master Instructor Village of Healing and Wellness, Canada; Ken Goh, Master Instructor President of Tai Chi Association Australia: Liu Kie Fee, Master Instructor Eastwood Community Tai Chi Club, Sydney; Angela Tian Zhu, Tai Chi and Qi Gong instructor, TCM practitioner, Sydney; Sue Chang, Tai Chi Master, Tai Chi Fitness Australia, Winston Hills, NSW, Australia; Warwick Poon, Qigong Adept, TCM practitioner, Melbourne, VIC, Australia; Susan Froehlich, LAc, Mosier, OR, USA; Ramel Rones, Master Instructor, Dana Farber Cancer Institute, Boston, USA; Lee Chan, Grand Master, Honorary Chairman of Korea Tai Chi Chuan Association, Seoul, Korea; Joseph Cheung, Tai Chi and Qigong Master, Director of Physical Arts, New York College of Health Professions, New York, USA; Hon Lee, Tai Chi Master, L,Ac, OMD, Hendon, VA. USA; Chi-hsiu D.Weng, Ph.D. Dean, College of Tai Chi, University of East-West Medicine, Sunnyvale, CA, USA.

## Nominations from Tai Chi and Qigong Committee Members

Paul Cheah, Tai chi and Qigong instructor, Eastwood Tai Chi Club, Sydney, Australia; Chris Findlater, Tai chi and Qigong practitioner, Eastwood Tai Chi Club, Sydney, Australia; Shane Kiely, Tai Chi and Qigong practitioner, University of Technology Sydney, Sydney, Australia; Stanley Lung, Tai Chi and Qigong practitioner, Eastwood Tai Chi Club, Sydney, Australia; Rena Czaplinska, PhD, Tai chi and Qigong practitioner, Sydney, Australia; Rosemary Yim, University of Technology Sydney, Sydney, Australia; Edwin Roy Franklin, Eastwood Tai Chi Club, Sydney, NSW, Australia; Lima Eid, Liverpool Tai Chi Club, Sydney, NSW, Australia; Vibeke Hansen, MAppSc, University of Newcastle, Australia; Yunho Choi, Lee Chan Tai Chi Chuan, Seoul, Korea; and Andy Huang, OMD, LAc, Chair of Public Safety Commission, Cupertino, CA, USA.
