# Supplementary material for: Accreditation Standard Guideline Initiative for Tai Chi and Qigong Instructors and Training Institutions
Source: Medicines (Basel). 2018 Jun 8;5(2):51. doi: 10.3390/medicines5020051 (PMC6023434; doi:10.3390/medicines5020051)
Supplement: Supplementary file 1 [file medicines-05-00051-s001.pdf]

Supplementary Materials: Accreditation Standard Guideline Initiative for Tai Chi and Qigong Instructors and Training Institutions

Byeongsang Oh, Albert Yeung, Penelope Klein, Linda Larkey, Carolyn Ee, Chris Zaslawski, Tish Knobf, Peter Payne, Elisabet Stener-Victorin, Richard Lee, Whanseok Choi, Mison Chun, Massimo Bonucci, Hanne-Doris Lang, Nick Pavlakis, Fran Boyle, Stephen Clarke, Michael Back, Peiying Yang, Yulong Wei, Xinfeng Guo, Chi-hsiu D. Weng, Michael R. Irwin, Aymen Elfiky and David Rosenthal

Certificate in Medical Tai Chi and Qigong Instructor (CMTQI) Policies and Procedures

Effective from 1 May 2018
Association Title: Medical Tai Chi and Qigong Association (MTQA)

Vision Statement: The vision of the Medical Tai Chi and Qigong Association (MTQA) is to advance global health through the practice of Tai Chi and/or Qigong.

Mission Statement: The mission of the association is to advance the practice of Medical Tai Chi and/or Qigong as a recognized area of practice within modern health care by both the health care community and the public.

Goals

1. to form and maintain an accreditation committee to develop and review accreditation standard guidelines for TQ instructors and training institutions within the context of integrative health care;
2. to establish and support *certified medical TQ instructors* (CMTQI) as emergent health care professionals to meet a work force need;
3. to inform the medical community as well as the general public, as to the credentialing and scope of practice of clinical TQ providers and the differentiation between traditional TQ instructors and those certified as medical TQ practitioners

Rationale

Evidence of benefits of TQ as a mindful, therapeutic system of exercise has been validated in research and practice, but TQ is under-utilized in modern health care, in part, due to limited awareness of the benefits of these therapeutic modalities among providers and the public. Utilization is further challenged by limited availability and recognition of a qualified work force to deliver these services. To date, there is no unifying regulatory body to sanction or certify clinically-trained TQ instructors and no higher education institutions in China nor Western countries to prepare this work force. Hence, a TQ instructor certification initiative was conceptualized with an understanding of the nature of complexity of TQ inherited from different principles, philosophies and schools of art and thought. The initiative is structured as an international collaboration of health professionals, integrative medicine practitioners and academics, Tai Chi and Qigong master instructors, and consumers. The results of this collaboration are policies and procedures for association governance and adjudication of a hierarchical model of standards of practice. This initial work should serve as a foundation for clarifying entry-level competence for educators, providers, and consumers as well as a scaffold for continuing education for a new category of health professional: Certified Medical Tai Chi Qigong Instructor.

Operational Definition of Terms Tai Chi - an ancient Chinese discipline of meditative movements practiced as a system of exercises — called also tai chi chuan.

Qigong-Qigong is an ancient Chinese system of postures, exercises, breathing techniques, and meditations. Its techniques are designed to improve and enhance the body's qi.

Certified Medical Tai Chi Qigong Instructor – one who has received certification of competence from the Medical Tai Chi and Qigong Association.

Acronyms

**TQ**	Tai Chi and/or Qigong
**MTQA**	Medical Tai Chi Qigong Association
**CMTQI**	Certified Medical Tai Chi and/or Qigong Instructor
**RPL**	Recognition of Prior Learning

Governance

1. The Association is an independent sanctioning body governed by an international, discipline-representative Advisory Board.

1.1. The Certificate of Medical Tai Chi and Qigong Instructor (CMTQI) is offered by the Medical Tai Chi and Qigong Association (MTQA) as a credential for Tai Chi and/or Qigong instructors to promote recognition by health practitioners and the public.

1.2. The CMTQI is awarded by the MTQA Council on the recommendation of the MTQA Certification Board. The role of the MTQA Certification Board is to:
1.2.1. Develop and publish curriculum guidelines.
1.2.2. Accredited courses.
1.2.3. Make recommendations to the MTQA Council regarding the award of the CRMTQI.

2. Eligibility

2.1. The CMTQI designation is available to suitably qualified members of MTQA.

2.2. Candidacy for the CMTQI is open to Tai Chi and/or Qigong instructors who meet the accreditation guidelines recommended by the MTQA

2.3. Applications for admission to the CMTQI must be made on the appropriate forms supplied by MTQA.
2.3.1. Certain designations may also have specific eligibility requirements as described within the relevant module/unit for each designation.

3. Training & Assessment

3.1. Tai Chi and/or Qigong
3.1.1. Training schools/ institutions run by qualified Tai Chi and/or Qigong instructors for training are recommended but not mandatory in order to apply for the CMTQI. However, the skills and proficiency of Tai Chi and Qigong applicants for certification will be reviewed and signed-off by suitably qualified Tai Chi and/or Qigong Masters from the community or from members of the Tai Chi and Qigong advisory committee of MTQA. This assessment will preferably be done in person at the relevant time after the application for certification is received or by recorded video if an in person assessment is not possible.
3.1.2. Logbook assessors and assessors of summative and formative assessments shall be members of the MTQA, including one health care professional.
3.1.3. The CMTQI Certification Board may approve other assessors who have equivalent expertise at its discretion.

3.2. Biomedical Science Module
3.2.1. Certificates of completion of biomedical and clinical medicine units issued by accredited approved tertiary institutions are acceptable or
3.2.2. Certificates of completion of biomedical and clinical medicine units issued by institutions

approved by the medical advisory committee of MTQA are acceptable.
3.2.3. Provisional guideline of biomedical science module: Each module requires a minimum of seven hours of biomedical science lecture and four hours of clinical application of TQ.

Anatomy and Physiology: The purpose of this course is to provide the TQ instructor with an understanding of the anatomy and physiology (structure and function) of the human body to enhance the ability to deliver TQ safely to individuals who have medical conditions. This course will be delivered by both online and face to face lectures including assessment of core competencies. The online program or face to face lectures (minimum 7 hours: provisional) covers the structure and function of the human body including cells, tissues and organs of the following systems: integumentary, skeletal, muscular, nervous and special senses. In addition, during the face to face lectures (4 hours: provisional), TQ instructors are expected to learn the following; a) TQ theory including yin yang and energy channel theory including Eastern philosophy, b) Clinical application of TQ, c) Potential risks and benefits of TQ, d) Evidence based TQ clinical practice, e) Clinical ethics, f) Doctor-TQ instructor communication skills, and g) Regulations of clinical health and public safety-practitioners adheres to professional, ethical, and legal requirements (ethics). At the end of the sessions, the competency of certified TQ instructors will be evaluated by multiple quizzes, case studies and demonstrations. All certified TQ instructors must be able to demonstrate a basic understanding of the human body and medical knowledge related to TQ education and training.

Other MTQA accredited courses in the specialized designations will be developed in collaboration with the Arthritis Foundation, Diabetic Foundation, Heart Foundation, Cancer Council and/or National Institute of Cancer, National Institute of Ageing, and tertiary academic institutions (mental health, stress management, obesity and lifestyle).

4. Requirements and Award

4.1. A CMTQI Certificate may be awarded to an instructor who is an MTQA member and who has fulfilled the following requirements:
4.1.1. Has completed MTQA accredited courses in the specialized designations in which he/she is qualified to practice.
4.1.2. Has completed either biomedical medicine and clinical medicine modules recommended by the MTQA or equivalent accredited modules.
4.1.3. Has completed all Tai Chi and/or Qigong practice requirements and submitted the portfolio evidencing attendance at accredited courses of specialist units

4.2. The list of accredited courses is maintained in the CRMTQI section of the MTQA website at: www.amtqa.org.

4.3. The CMTQI Certification Board may, at its discretion, recognize a candidate's training and experience as meeting some or all of the requirements for CMTQI certification. Candidates wishing to apply for recognition of prior learning must do so using the form available on the MTQA website at: www.amtqa.org.

4.4. The CMTQI Certificate will list the specialist designation awarded.
4.5. The CMTQI may be jointly awarded with endorsement from another Association or Society.

5. Certification Standards

5.1. Evaluative domains of mastery include:

a. technical performance skill in style(s) and form;
b. content knowledge: clinical anatomy, physiology, energy theory, applied research, and ethics in practice;
c. teaching mastery;
d. scholarly contributions to the discipline of clinical TQ practice; and
e. service to the discipline, the individual, one's community, and our global society.

5.2. Assignment of certified Clinical Instructor level is guided by Bloom's hierarchy of learning. Technical performance is prerequisite and must be verified by a master-level instructor (equivalent to Level III or Level IV instructor) within respective styles of TQ.

Level I certifies technical mastery of style(s) and form (≥50 minimum hrs of instruction) plus a portfolio of ≥150 hrs of applied education. Applied education to include basic knowledge of integration of Eastern Energy theory (≥10 hrs) and Western health care (≥10 hrs) including a basic understanding of human anatomy and physiology pathogenesis and precautions and considerations for TQ exercise supervision of individuals with common clinical conditions (≥20), as well as class management to assure safety and maintenance of dignity of participating students/clients (≥5 hrs), use of research at a consumer level (≥5); a minimum of 30 hours of teaching clinical populations, and statement of willingness to adhere to standards of ethical practice (≥5 hrs).

Note: technical training credit is limited to 50 hrs of the total learning experience; teaching credit is limited to 30 teaching hrs. There is a required minimum of 10 hrs of human anatomy and physiology or equivalent and a required minimum of 10 hrs of instruction in Energy theory or equivalent. Teacher training credit of 5 hrs or equivalent credit along with 5 hrs of training in practice ethics is required. The remaining 90 experience/training credited hrs may include online or direct learning, seminars/conference attendance or presentation, discipline-related course work, and/or scholarly activity that advance one's discipline-related skills and/or contributes to advancement of the discipline as well as a maximum of 20 hrs of service credits. Additionally, candidates must present a plan for continuing education. (credited 5 hrs for this product)

Level II certifies that all components of Level I are met plus a more in depth understanding of special considerations for supervision and exercise progression of individuals with common clinical conditions and applied use of emerging research to guide best practice and evidence of a minimum of 100 teaching hrs of individuals with common clinical conditions.

Level III certifies that all requirements of Level II instructor are met plus a minimum of 200 teaching hours of individuals with common clinical conditions as well as evaluative skills as demonstrated in applied curriculum. Instructors at this level will be engaged in training instructors (train the trainer) as well as advancing awareness of the discipline at a regional level.

Level IV certifies that all requirements of Level III are met plus a minimum of 500 teaching hrs as well as engagement in scholarly activity that advances the profession. Scholarly activity may be in the form of participation in collaborative clinical research, validation of theory, development of educational curriculum, offering of professional presentations, authoring of scholarly publications, and service to the profession. At this level, the Instructor should have a national or international presence within the discipline.

Table 1. Certification Standards.

Level/criterion	I	II	III	IV
Style/form technical skill	Documented and verified by expert teacher	Documented	-	-
Direct teaching hrs	≥30 hrs	≥100	≥200	≥500
Knowledge of anatomy and physiology*	Basic 10 hrs	Applied	Applied	applied
Knowledge of Energy theory**	Basic 10 hrs	Applied	Applied	Applied
Research	Consumer-level	Applied-level	Evaluative--level	Advanced - level
Class management	Basic	Applied	Model -Teaching	Teaching mastery

Ethical Practices (affective domain)	Model healthy behaviors Signed Code of conduct	Honor the individual	Instill ethical practice in others	Practice for the greater good
Maintain Privacy & Dignity of students	basic	basic	basic	basic
Continuing Education	Recertification every 2 yrs ≥10 hrs.	Recertification every 2 yrs ≥20 hrs	Recertification every 2 yrs ≥20 hrs	Life-long
Service Level	Local	Local and/or Regional	Regional and/or National	National and/or International

* Credit for documented life experience and Medical, nursing, allied health, athletic training, chiropractic, exercise or rehabilitation specialist, and naturopathic professional education will be given where appropriate. ** Credit for documented life experience as a certified energy practitioner, acupuncturist, doctor of Chinese Medicine.

5.3. Evidence of meeting standard criteria will be presented as a documented portfolio. A portfolio rubric and sample portfolios will be made available by the Association to aid in portfolio construction.

5.4. Service may take many forms including but not limited to health fair presentations, organizing local, regional, national, or international conferences, Association or discipline-related committee work or maintenance of discipline-specific electronic communications such as web pages or blogs.

5.5. Scholarly work may take many forms including but not limited to scholarly presentations at local or regional seminars (basic-level), discipline-related presenting at national or international professional seminars (advanced-level), article submissions to magazines (basic-level), peer-reviewed scientific publications (advanced level), text authoring, active engagement in research to validate theory.

5.6. Evidence of active teaching and engagement in and a plan for continued learning are conditions for recertification.

6. Recertification

6.1. Recertification will occur at three-yearly intervals after the initial certification, on presentation of the requisite evidence demonstrating that the candidate has met Practice Requirements and Continuing Professional Development Requirements (CPD).
6.2. To achieve recertification the candidate must:
6.2.1. Complete at least 100% of the original CPD portfolio requirements per annum as specified in the relevant unit syllabus. At least 50% of these CPD must be clinically indicated.
6.2.2. Complete at least 50% of the relevant CPD each year for each specialist designation. The maximum number of CPD points required per year for each candidate is a total of 20 points.
6.2.3. Continue to fulfill the conditions for Eligibility and Admission to the CMTQI.

6.3. Recertification will only be given for those specialized units where recertification requirements have been met.

6.4. The MTQA/CMTQI Certification Board may, at its discretion, suspend the CMTQI for a period of up to 2 years where the clinician demonstrates circumstances which prevent him/her from meeting recertification requirements.

7. Currency of Qualification

7.1. An instructor's CMTQI will cease to be current if he or she:
7.1.1. Ceases to be a member of the MTQA; or
7.1.2. Fails to meet the requirements for recertification.

7.2. In the event that a person's CMTQI ceases to be current, it may be restored within 12 months by fulfilling the conditions to apply for the CMTQI, meeting the Recertification Requirements and meeting any other conditions required by the MTQA/ CMTQA Certification Board. Thereafter it may be restored only by meeting all requirements as detailed in the preceding clauses.

8. Reconsideration

8.1. Candidates may challenge the decision of the MTQA /CMTQI Certification Board should write in the first instance to the Chair of the MTQA /CMTQI Certification Board within one calendar month of the date on which notification of the result was sent, stating the facts which the Chairman should consider when determining whether or not to uphold the original decision.

8.2. Applications for reconsideration will be acknowledged when they are received at the MTQA office.

8.3. Applications for reconsideration will be considered and determined expeditiously by the Chairman of the MTQA/CMTQI Certification Board. In determining the matter, the Chairman has discretion to use any available information, but is not required to consider any information other than that provided by the applicant.

8.4. Applicants for reconsideration will receive formal written notification of the results of their application.

8.5. Applications for Reconsideration must be made on the prescribed form and be accompanied by full payment of the prescribed fee.

9. Appeals

9.1. An application to appeal a decision of the MTQA/CMTQI Certification Board will only be accepted once the Reconsideration procedures prescribed in these regulations have been exhausted.
